# Calibration of a Low-Cost Methane Sensor Using Machine Learning

**DOI:** 10.3390/s24041066

**Published:** 2024-02-06

**Authors:** Hazel Louise Mitchell, Simon J. Cox, Hugh G. Lewis

**Affiliations:** Computational Engineering and Design Group, Faculty of Engineering and Physical Sciences, University of Southampton, Southampton SO17 1BJ, UK

**Keywords:** methane, machine learning, sensor, calibration

## Abstract

In order to combat greenhouse gas emissions, the sources of these emissions must be understood. Environmental monitoring using low-cost wireless devices is one method of measuring emissions in crucial but remote settings, such as peatlands. The Figaro NGM2611-E13 is a low-cost methane detection module based around the TGS2611-E00 sensor. The manufacturer provides sensitivity characteristics for methane concentrations above 300 ppm, but lower concentrations are typical in outdoor settings. This study investigates the potential to calibrate these sensors for lower methane concentrations using machine learning. Models of varying complexity, accounting for temperature and humidity variations, were trained on over 50,000 calibration datapoints, spanning 0–200 ppm methane, 5–30 °C and 40–80% relative humidity. Interaction terms were shown to improve model performance. The final selected model achieved a root-mean-square error of 5.1 ppm and an R^2^ of 0.997, demonstrating the potential for the NGM2611-E13 sensor to measure methane concentrations below 200 ppm.

## 1. Introduction

Atmospheric methane (CH_4_) plays a significant role in global warming. Methane is released into the atmosphere through a combination of natural sources (e.g., peatlands), human activities (e.g., agriculture, pipeline leaks) and the release of trapped stores due to rising global temperatures (e.g., permafrost melt) [1].

A variety of approaches exist for monitoring methane emissions. Beginning with the greatest coverage, satellites can provide global methane concentration data but are limited in either temporal or spatial resolution [2]. Existing satellites are also limited in the terrains over which methane concentration measurements can be taken; data cannot be provided for sea, snow or marshland. Higher-resolution surveys may be carried out by aircraft or ground teams but these methods require additional labour for each new dataset collected.

Autonomous local sensors, such as weather stations, address these issues, continuously collecting data at high frequencies, in some cases every few seconds. However, many weather stations are permanent standalone installations, based in cities, and, as such, have limited spatial coverage. To compensate for this, wireless sensor networks and “Internet of Things” (IoT) devices are increasingly used to augment datasets, especially in the field of air pollution monitoring, adding additional data collection sites [3,4,5,6,7,8]. IoT devices are typically powered by batteries or small solar panels; therefore, minimising power consumption is crucial [9]. Much of the value of wireless sensor networks lies in their coverage and scalability, so enabling large fleet sizes by minimising the individual device cost is also a key consideration [5]. As such, the sensors included in these devices should accommodate these requirements.

Many categories of methane sensors have been developed: optical, capacitance-based, calorimetric, resonant, acoustic-based, pyroelectric, metal oxide semiconductor (MOS), and electrochemical [1,10,11]. Of these types, MOS sensors show particular potential for compact, low-power and low-cost applications, but additional steps must be taken to improve their performance in outdoor settings [10,12,13,14,15,16].

Machine learning techniques are often employed to improve the usability of gas sensor data, by addressing either the selectivity of the sensor or the calibration accuracy [11,13,17]. Classification algorithms, such as support vector machines and neural networks, have been shown to improve the ability of both individual sensors and sensor arrays to identify specific target gases or gas mixtures [18,19,20,21,22,23]. This approach holds promise for a variety of emerging applications, ranging from disease diagnosis from the gas composition of human breath [22] to identifying specific sources of air pollution in urban environments. Regression machine learning can be used to calibrate the output of gas sensors in varying environmental conditions—i.e., target gas concentration, temperature and air humidity—and to offset long-term sensor drift [24].

The Figaro NGM2611-E13 (Figaro, Rolling Meadows, IL, USA) is a low-cost methane detection module based around the TGS2611-E00 MOS sensor [25]. The manufacturer provides sensitivity characteristics for methane concentrations above 300 ppm [26], but lower concentrations are typical in outdoor settings.

Several authors have investigated methods for calibrating this sensor at lower methane concentrations [14,15,16]. Results are consistently encouraging, with strong correlation between calibrated sensor output and true methane concentration achieved in all of these studies.

Van den Bossche et al. [14] calibrated a TGS2611-E00 methane sensor across 15–30 °C, 40–80% relative humidity and 2–9 ppm methane. Methane concentration was recorded using a Picarro G2301 Cavity Ringdown Gas Analyzer (Picarro, Santa Clara, CA, USA). For this limited methane concentration range, a linear fit was assumed for the sensor calibration, with temperature and humidity compensation applied separately. Within this range, a systematic error of −1.0 ppm and a variable error of ±1.7 ppm in estimated methane concentration were achieved. It should be noted that a linear fit cannot be assumed for wider ranges of methane concentration; an exponential relationship is visible in the 300–10,000 ppm range published by the manufacturer [26].

Bastviken et al. [16] investigated a calibration approach for the NGM2611-E13 using estimated background methane concentration in a chamber, followed by the injection of methane up to 719 ppm. They tested 15 model equations with the collected chamber data, achieving strong correlation between the model output and true methane concentration (R^2^ = 0.99–1.00) and low error (RMSE = 9.8–20) over the full tested concentration range up to 719 ppm.

These existing studies share two main limitations: (1) expensive reference instruments are used to measure methane concentration during sensor calibration, and (2) potential interactions between temperature, humidity and methane concentration are not addressed.

Collier-Oxandale et al. [15] calibrated the Figaro TGS 2600 by co-deploying sensors with reference-grade instruments in field deployments, during which methane concentrations remained below 6 ppm. Variable correlations were achieved between the sensors and reference measurements (R^2^ = 0.625–0.812) for the best-performing model. Terms representing the interaction between temperature and methane concentration were considered, but all of the models which contained such a term also included a time-based term. As recurring diurnal emission cycles are not universal and will vary by site, time-based predictor variables are not applicable to pre-deployment sensor calibration.

This study presents an alternative calibration approach using 200 ppm methane-in-air calibration gas and machine learning models. The calibration conditions span 5–35 °C and 40–85% relative humidity. A range of nonlinear models derived from the sensor response to varying methane concentration, temperature and humidity were formulated and tested, including models with interaction terms. A calibration validation method using 200 ppm methane in air is also presented.

## 2. Materials and Methods

### 2.1. Methodology Overview

**Figure 1:**
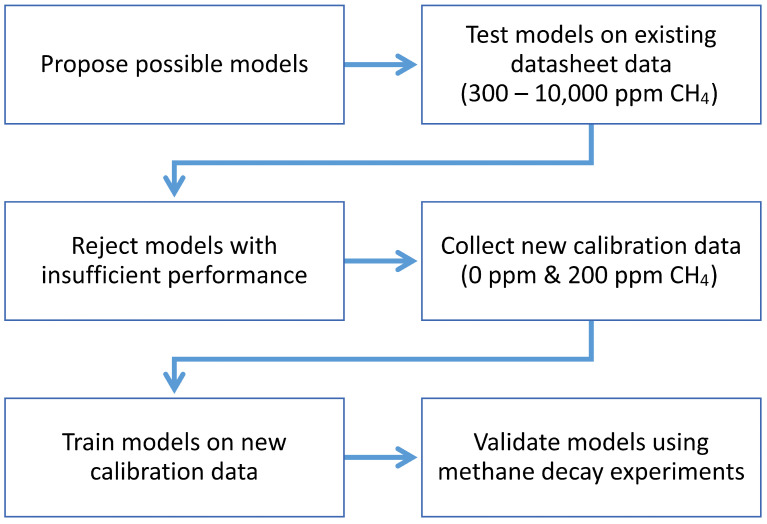
Methodology flowchart.

An overview of the methodology approach is depicted in Figure 1. These stages are explained in more detail in the following subsections.

### 2.2. Methane Sensor Characteristics

The NGM2611-E13 is a module designed for use in natural gas leak detectors. This sensor module was selected for investigation in this study as it meets the requirements of sensors for typical IoT devices; the sensor is affordable (<GBP 30 per unit), compact (27 × 12.5 × 14.1 mm), and has an operating voltage of 5 V.

The sensing element in the module is a TGS 2611-E00. The NGM2611-E13 and TGS 2611-E00 datasheets provided by Figaro include characterisation data for the methane sensor across a range of temperature (−10–40 °C), relative humidity (35–95%) and methane concentration (300–10,000 ppm) conditions [25,26]. The sensor response is described by the ratio $R_{S}/R_{0}$, where $R_{S}$ is the measured sensor resistance and $R_{0}$ is the sensor resistance in 5000 ppm of methane at 20 °C, 65% relative humidity (RH).

**Figure 2:**
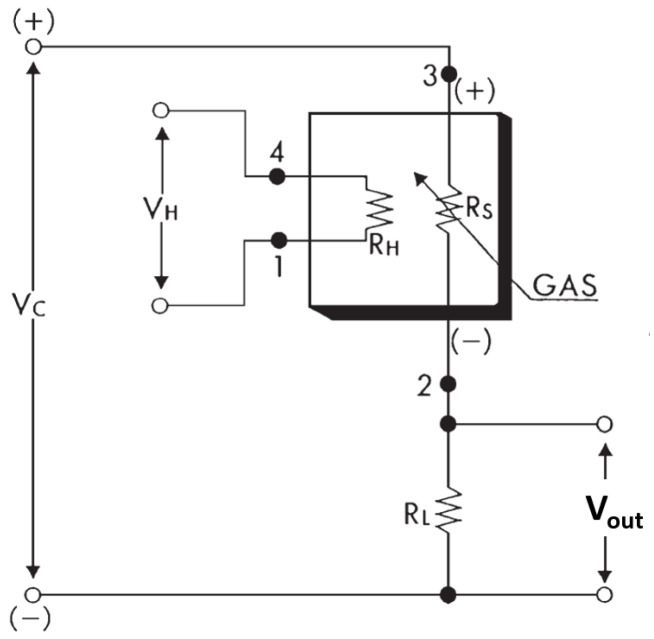
TGS 2611-E00 methane sensor circuit, adapted from [25].

The NGM2611-E13 sensor module comprises a methane sensing element ($R_{S}$), a current limiting resistor ($R_{L}$) and a heating element ($R_{H}$). The sensing element is arranged in a potential divider circuit (Figure 2), such that the sensor output voltage, $V_{out}$, can be calculated as:

(1) $$V_{out}=\left(V_{C}\cdot R_{L}\right)/\left(R_{L}+R_{S}\right)$$

where $V_{C}$ is the supply voltage for the sensor circuit (i.e., 5V), $R_{S}$ is the measured sensor resistance and $R_{L}$ is the value of a fixed resistor (given as 10.0 kΩ±1% for standard test conditions in the sensor datasheet).

**Figure 3:**
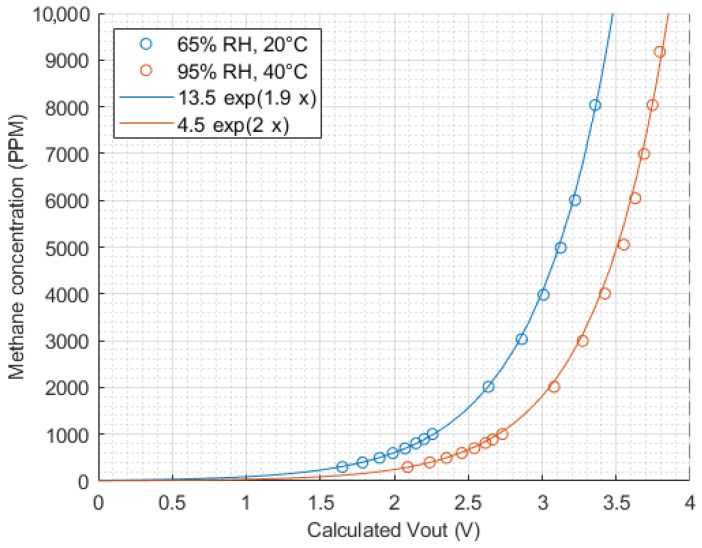
Sensor output vs. methane concentration (extracted from Figaro datasheets and converted from sensor resistance ratio to $V_{out}$).

**Figure 4:**
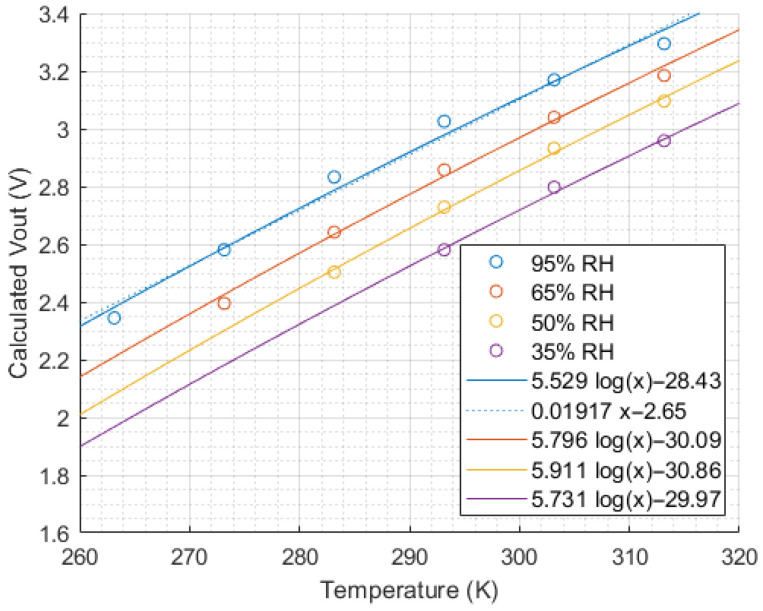
Sensor output vs. temperature at 5000 ppm methane concentration (extracted from Figaro datasheets [26] and converted from sensor resistance ratio to $V_{out}$).

**Figure 5:**
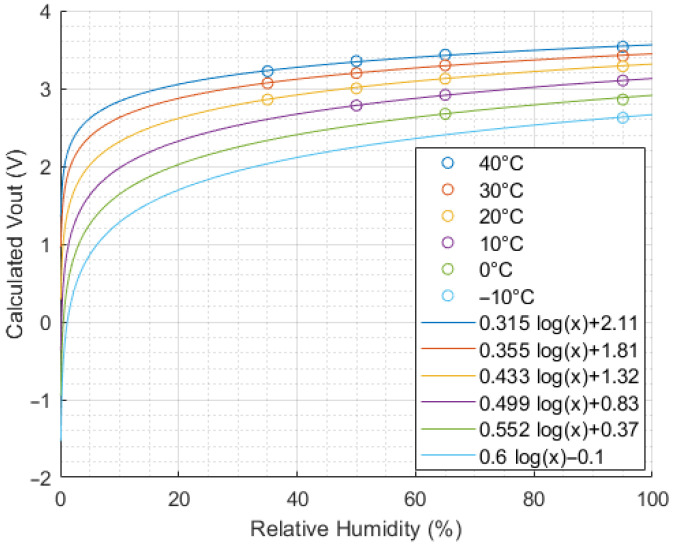
Sensor output vs. humidity at 5000 ppm methane concentration (extracted from Figaro datasheets [26] and converted from sensor resistance ratio to $V_{out}$).

Using this equation and data extracted from the Figaro datasheets, Figure 3, Figure 4 and Figure 5 were constructed to visualise the influence of methane concentration, temperature and relative humidity on the sensor output voltage.

**Figure 6:**
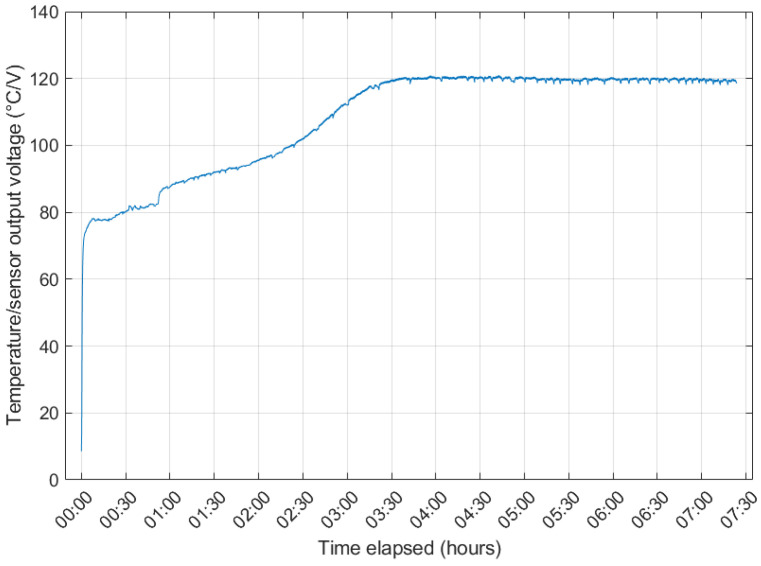
Example of the NGM2611-E13 sensor warm-up behaviour, showing stabilisation after around 4 h.

In early chamber experiments, an initial warm-up period was identified for the NGM2611-E13 (Figure 6). In normal operation, a linear relationship is observed between the sensor output voltage and the air temperature. For up to 4 h after being powered, the sensor output does not follow this trend, presumably because the heating element in the sensor takes time to reach thermal equilibrium with the surrounding air. To avoid disrupting the calibration data, this warm-up period was excluded from the calibration data for the experiments detailed later.

### 2.3. Model Form

In this study, we consider a nonlinear regression machine learning approach. Compared with more complex approaches, such as neural network models, nonlinear regression models are easier to interpret in terms of how the output is affected by each predictor. Nonlinear regression models also require less training time and smaller training datasets once the model form has been selected.

In order to determine a suitable form for the methane sensor calibration model, we first consider the influence of methane concentration, temperature and humidity on the sensor output voltage.

From Figure 3, we can derive a relationship between $V_{out}$ and methane concentration of the form:

(2) $$M=C_{1}\cdot e^{C_{2}\cdot V_{out}}$$

where *M* is the methane concentration in ppm, $C_{1}$ and $C_{2}$ are temperature- and humidity-dependent constants and $V_{out}$ is the sensor output voltage in *V*. This forms the starting point for our model.

Figure 4 and Figure 5 show a logarithmic relationship between the sensor output voltage and temperature or humidity. For the same methane concentration, a higher temperature or higher humidity will increase the sensor output voltage. Note that a linear fit may be a sufficient approximation for small temperature variations, as shown by the dashed line in Figure 4, but a logarithmic fit provides a higher coefficient of determination and smaller residuals, so it was selected for use in this study.

Incorporating additional terms for the effect of temperature, humidity and interactions between the variables, a general model form is proposed:

(3) $$M=C_{1}+C_{2}\cdot e^{C_{3}\cdot V_{out}-C_{4}\ln\left(T+\alpha \right)-C_{5}\ln\left(H+\beta \right)-C_{6}\ln\left(\left(T+\alpha \right)\left(H+\beta \right)\right)-C_{7}\ln\left(\left(T+\alpha \right)V_{out}\right)-C_{8}\ln\left(\left(H+\beta \right)V_{out}\right)}$$

where *M* is the methane concentration in ppm, $C_{1}$–$C_{8}$ are constants to be optimised by the fitting algorithm, α and β are offsets to be applied to the temperature and humidity terms, respectively, T is temperature (°C), H is relative humidity (%) and $V_{out}$ is the methane sensor output voltage (V).

### 2.4. Initial Model Testing and Shortlisting

**Figure 7:**
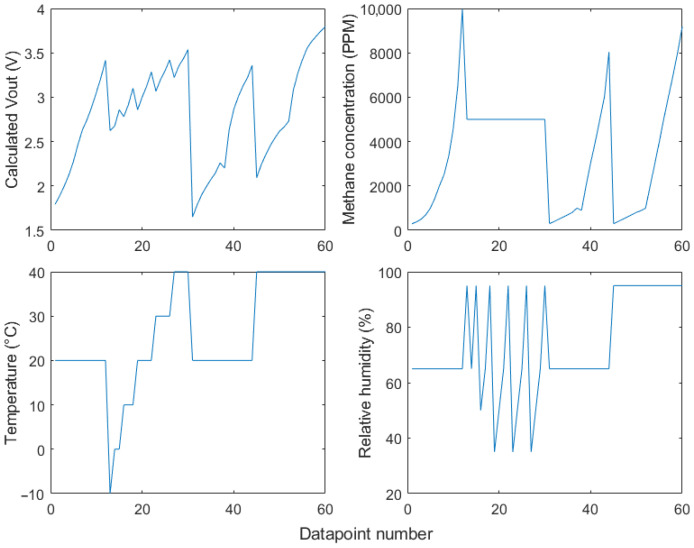
Training data extracted from the Figaro datasheets [26] and converted from sensor resistance ratio to $V_{out}$ where required.

The data shown in Figure 3, Figure 4 and Figure 5 were compiled into a training dataset (Figure 7) to determine an appropriate non-linear model form. This dataset was used to test a series of sensor calibration models of increasing complexity, starting with a calibration based only on the sensor output voltage and gradually adding compensation for temperature and humidity. “Complexity” is defined as the number of constant terms + the number of variable terms used in the model. These models are listed in Table 1.

Nonlinear regression models are trained by taking an equation that relates a continuous response variable to one or more predictor variables and adjusting the coefficients of the equation to optimise its fit to the provided training data. In this case, the response variable is methane concentration and the predictor variables are sensor output voltage, temperature and relative humidity. Each model was optimised using the MATLAB “fitnlm” function [27]. Initial estimates were set to 0 for all coefficients. A termination tolerance of 1×10 ^−8^ and a limit of 200 iterations were used.

### 2.5. Collection of Calibration Data

**Figure 8:**
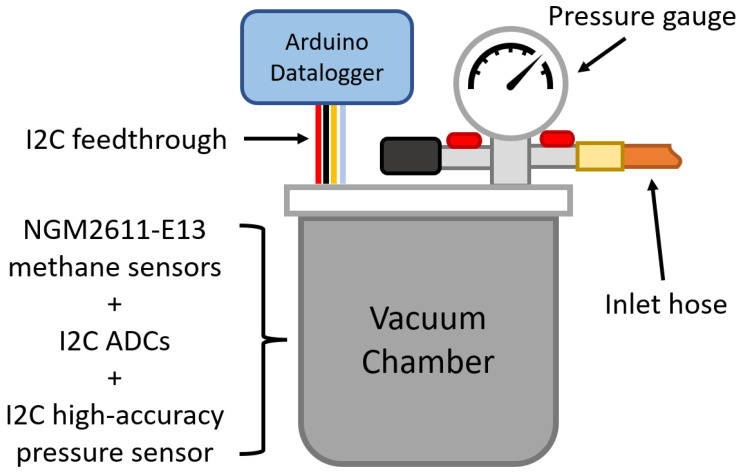
A diagram showing the vacuum chamber setup.

Four NGM2611-E13 methane sensors were placed inside a vacuum chamber set up as shown in Figure 8. The output voltage and internal reference voltage of the sensors were measured using a pair of Velleman 4-channel ADS1115 16-bit Analog-to-digital converter I²C modules (Velleman, Gavere, Belgium) [28]. An ICP-10125 high-accuracy pressure sensor (InvenSense, San Jose, CA, USA) [29] measured air pressure and temperature inside the chamber. An Arduino datalogger provided power to the sensors inside the chamber and recorded the sensor readings to a micro SD card every 2–3 s (Arduino, Somerville, MA, USA).

**Figure 9:**
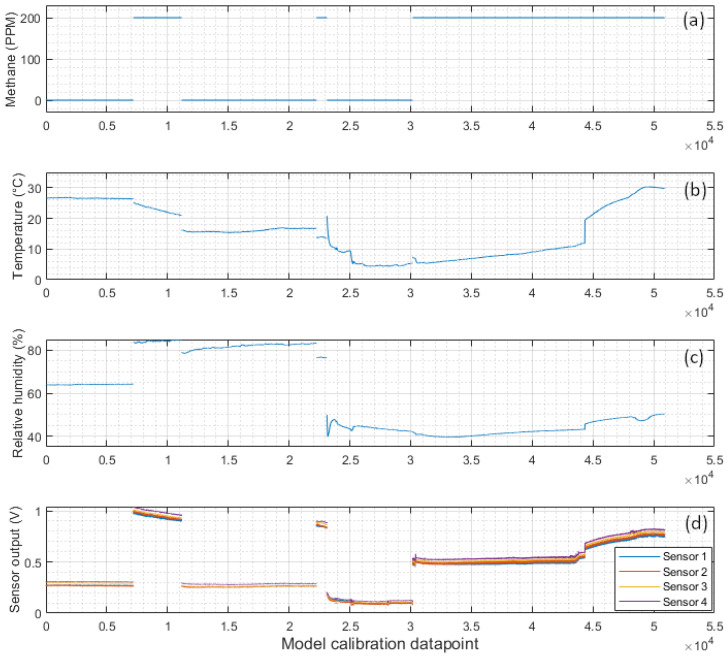
A summary of the data used to calibrate the methane sensors: (**a**) methane concentration, (**b**) air temperature, and (**c**) relative humidity inside the chamber, and (**d**) the recorded voltage output of the NGM2611-E13 sensors in the chamber.

A vacuum pump connected to the inlet hose was used to evacuate the air from inside the chamber whilst the sensors were powered off. Then, 200 ppm methane in air calibration gas [30] was used to refill the chamber to atmospheric pressure. Over the course of up to 24 h for each methane concentration level, sensor readings were recorded by the external Arduino datalogger. The experiment was repeated using calibration air (20.9% oxygen balanced in nitrogen) and at low, moderate and high temperatures and relative humidity. The vacuum chamber was placed in an ice bath to gather sensor readings at low temperatures. A higher humidity was created in the vacuum chamber by placing a small dish of ice cubes in the base of the chamber. An overview of the calibration data collected is shown in Figure 9.

### 2.6. Model Testing on New Calibration Data

Using the calibration data, the model equations carried over from the tests on the data from the sensor datasheets were optimised using the same process as before. As the four methane sensors showed very similar responses (Figure 9), the models were trained on only one of the sensors.

To investigate if improved model performance could be achieved, manually adjusted variations of Equation (20) with different temperature and humidity offsets were also tested. Table 2 lists two variations: Equation (21) is the model which yielded the lowest RMSE and highest coefficient of determination (R^2^), and Equation (22) is a variation of the same form without a humidity offset applied.

### 2.7. Methane Decay Experiments

**Figure 10:**
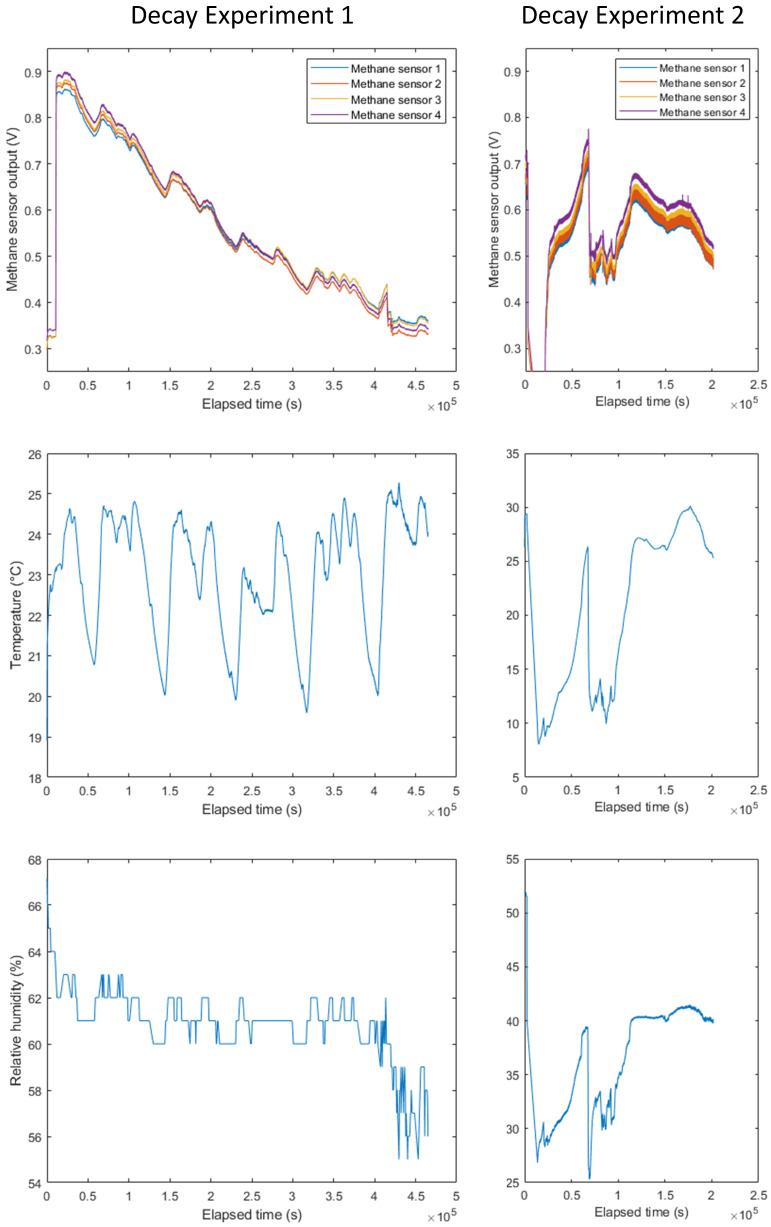
Recorded methane sensor output voltage, chamber temperature and relative humidity throughout the methane decay experiments.

To check the validity of the calibrated models and identify any model overfitting, two experiments with a decaying methane concentration were performed. The sensors were placed inside a sealed vacuum chamber. Then, 200 ppm methane in air calibration gas was injected into the chamber to raise the methane concentration, and the second inlet valve was opened to equalise the pressure inside the chamber. This process was repeated until the sensor readings no longer rose when additional calibration gas was injected—i.e., when the methane concentration within the chamber had reached approximately 200 ppm. The chamber inlets were then opened, allowing the methane concentration inside the chamber to steadily decay towards the external ambient concentration. This experiment was repeated twice: the first experiment was run in ambient conditions (19–25 °C) over 4 days; the second utilised an external ice bath to vary the chamber temperature over a wider range (8–30 °C) and a shorter period of 2 days (Figure 10).

A valid model would be expected to show the following features when applied to each decay experiment:

- A steady initial methane concentration below 50 ppm before the calibration gas was injected.
- A peak concentration of around 200 ppm methane.
- A steady decline in methane concentration following the peak.

## 3. Results

### 3.1. Initial Model Testing and Shortlisting

Table 3 summarises the performance of each of the model equations fitted on the training data shown in Figure 7. This table was used to determine which equations to pursue in the following calibration experiments.

Model Equations (1)–(4) and (7)–(10) were rejected, having R^2^ < 0.9. Equations (13) and (14) were more complex than Equation (12) but had a higher root-mean-square error (RMSE) and were therefore also rejected. Equation (19) performed similarly to Equations (15) and (17) (RMSE = 198, vs. RMSE = 196), warranting further investigation. Models which included the $C_{1}$ term generally presented slightly a higher RMSE than the corresponding equation of the same form without the $C_{1}$ term, but showed better performance at lower methane concentrations; as such, Equations (16), (18) and (20) were retained for further testing.

### 3.2. Methane Sensor Calibration

Model Equations (5) and (6) performed far worse on the calibration data than on the data extracted from the NGM2611-E13 datasheets, with lower R^2^ values (Table 4) than before (Table 3). Equation (5) overestimated the methane concentration for all 0 ppm methane datapoints and overcompensated for high temperatures at 200 ppm methane.

**Figure 11:**
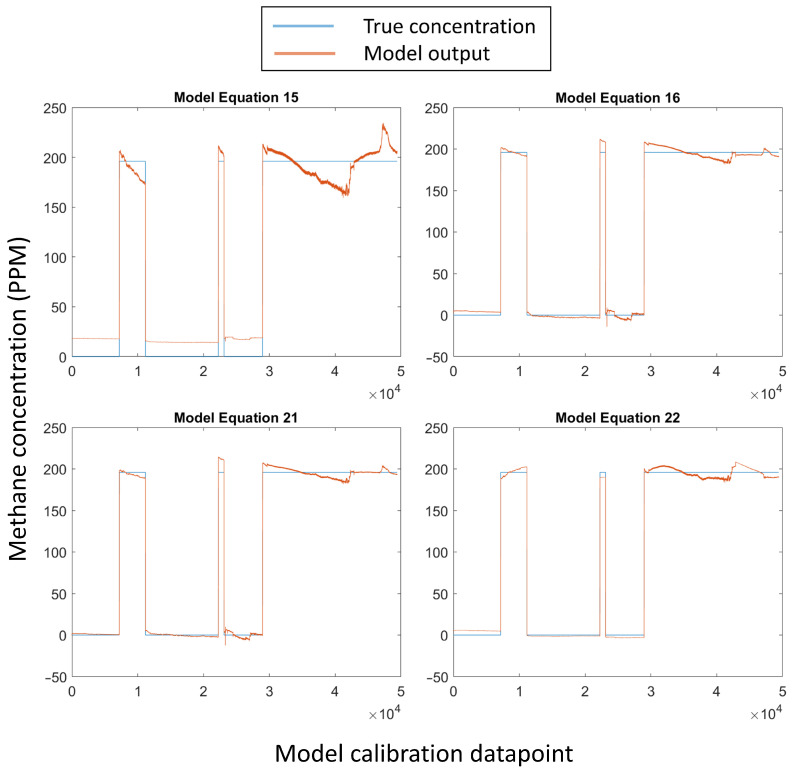
A subselection of the trained models (Equations (15), (16), (21) and (22)). Plots showing all of the trained models are included in Appendix A.

Equations (11), (15), (17) and (19) showed similar fits (Figure 11). All four of these models overestimate the methane concentration at 0 ppm and show inadequate temperature compensation at 200 ppm.

Equation (12) fitted similarly to Equations (11), (15), (17) and (19), with the main difference being that Equation (12) predicts negative values for the 0 ppm datapoints at moderate temperatures (around 15 °C) and high humidity (above 75%).

Of the models tested, model Equations (16), (18) and (20) demonstrated the best performance, with high R^2^ (0.997) and low RMSE (≈5.1 ppm) values (Table 4). These models also show fairly uniform fitting across the whole range of the calibration data (Figure 11). The coefficients for model Equation (16) are listed in Table 5.

Equations (21) and (22) showed marginally improved performance over Equations (16), (18) and (20), with a higher R^2^ (0.998) and lower RMSE (4.5 ppm and 4.79 ppm, respectively) but at the cost of much higher model complexity (Table 4).

### 3.3. Methane Decay Experiments

The rapid temperature change at around 0.7 × 10^5^ seconds in the second decay experiment manifested as a spike around the same time in the estimated methane concentration for most of the model equations.

Equations (5) and (6) perform reasonably well for the first decay experiment but both underestimate the peak methane concentration in the second experiment.

Equations (11) and (12) overestimate the peak methane concentration in both decay experiments and also estimate different ambient methane concentrations at the start of the two experiments.

**Figure 12:**
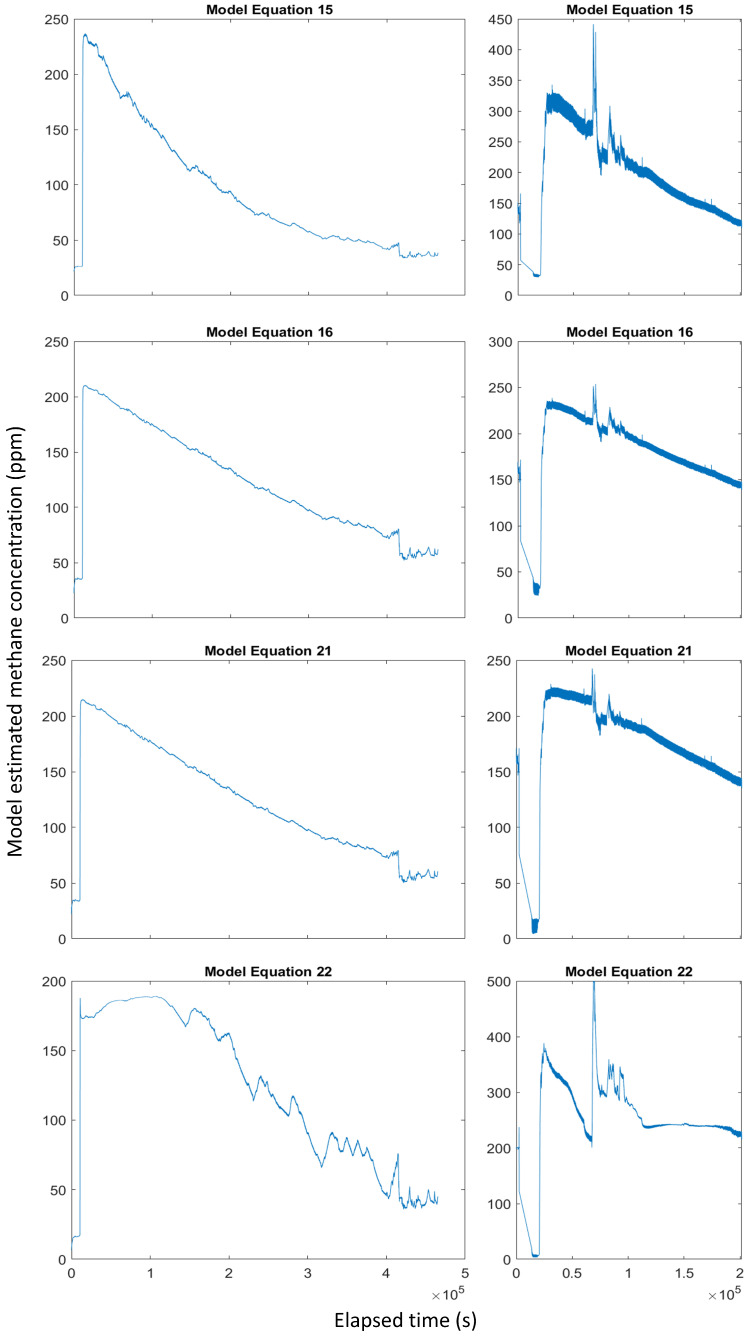
Estimated methane concentration from the decay experiment using model Equations (15), (16), (21) and (22). Plots showing all of the trained models are included in Appendix B.

Equation (15) poorly compensates for temperature and humidity variations (Figure 12).

Equations (17) and (19) overestimate the peak methane concentration in both decay experiments.

**Figure A5:**
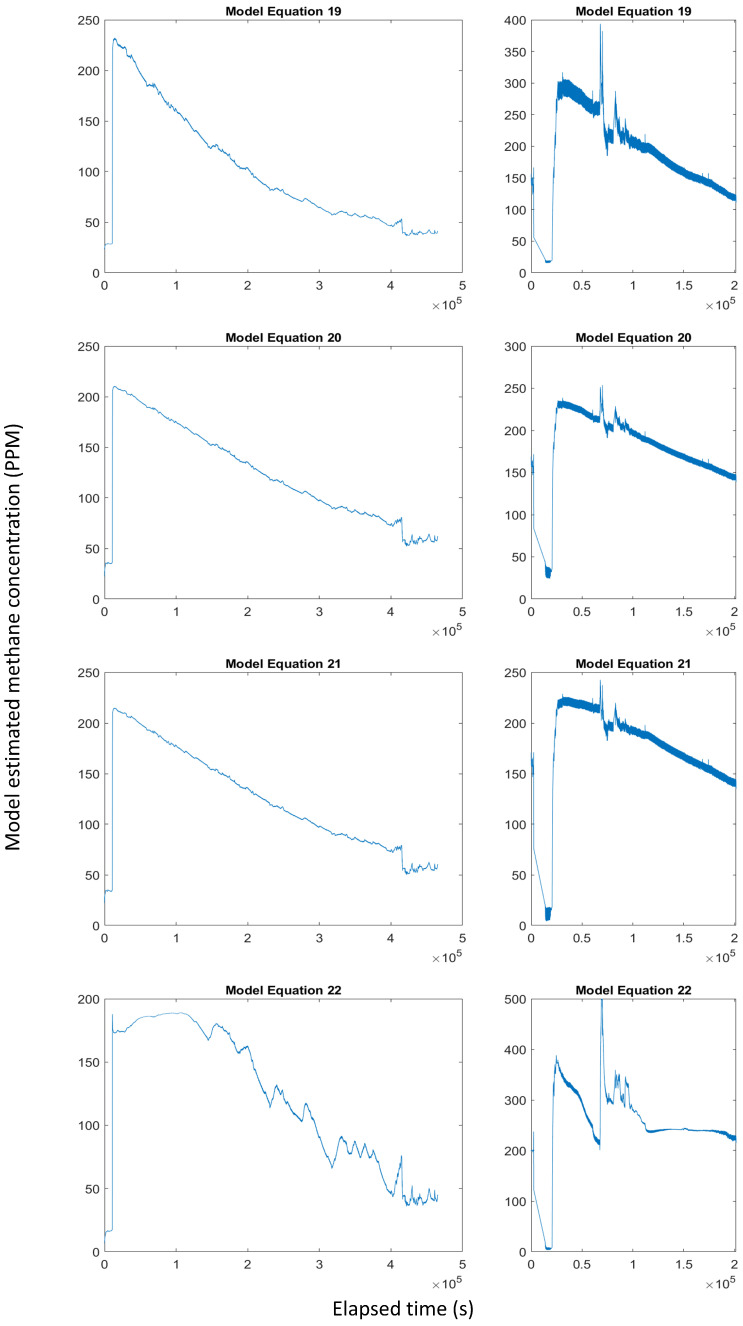
Estimated methane concentration from the decay experiment using model Equations (19)–(22).

Model Equations (16), (18), (20) and (21) provided the best performance for the decay experiments (Figure A5). For the first decay experiment, all four models estimate an initial methane concentration of around 30 ppm, a peak concentration of around 210 ppm and a smooth decay curve. Similarly, in the second decay experiment, the models estimate an initial methane concentration below 50 ppm, a peak concentration of around 225 ppm and an appropriate curve shape.

When applied to the decay experiment data, model Equation (22) displays clear signs of overfitting (Figure 12). Despite the high R^2^ (0.998) and low RMSE (4.79 ppm) of the model trained on the calibration dataset, in both decay experiments, the model fails to compensate for variations in temperature and humidity. This is most obvious from the sawtooth-shaped peaks in the first decay experiment, which correspond to peaks in the chamber temperature. The model also severely overestimates the methane concentration throughout the second decay experiment.

## 4. Discussion

This study aimed to investigate the practicality of using machine learning to calibrate low-cost methane sensors at lower methane concentrations than required for their typical applications.

The greatly improved performance of model Equation (11) (RMSE = 19.2, R^2^ = 0.962) compared to Equations (5) and (6) (RMSE = 55 and 35.6, R^2^ = 0.684 and 0.868, respectively) shows that both temperature and relative humidity need to be accounted for when calibrating the NGM2611-E13 sensor at methane concentrations below 200 ppm. The strong correlation of Equation (11) with true methane concentration also validates the approach of using an equation of this form to model the sensor readings.

Additional interactions between temperature, relative humidity and methane concentration are expected to affect the output voltage of the methane sensor. More complex machine learning models containing additional terms can capture these interactions and reduce the error in the model. However, increased complexity carries a greater risk of overfitting, which can render a model useless for making predictions from new data. The risk of overfitting can be reduced by expanding the training data or using more intensive model validation, both of which increase the computing load when training the model. Therefore, for any machine learning model, a balance exists between model robustness and detail.

In this study, increased model complexity broadly correlated with improved model performance: i.e., lower RMSE and higher R^2^. However, diminishing returns in increasing model complexity are also clearly shown by the results in Table 4. The best performing models: Equations (16), (18), (20) and (21), showed very similar performance (RMSE = 4.5–5.1 ppm, R^2^ = 0.997–0.998). The model with the next lowest RMSE was Equation (12) with RMSE = 14.2 ppm, which is almost three times that of the top four models. For model Equations (15)–(20), including an offset term, $C_{1}$ reduced the RMSE of each model by around 10 ppm. The additional improvement of Equations (16), (18), (20) and (21) over Equation (12) can be attributed to the inclusion of a temperature and sensor output voltage product term or a relative humidity and sensor output voltage product term. It is unsurprising that the effect of including either one of these terms, or both at the same time, is similar because relative humidity is roughly inversely proportional to air temperature in a sealed volume. Therefore, either term will effectively accommodate the same effect of the environmental conditions in the chamber.

Despite containing almost twice as many terms, model Equation (21) (RMSE = 4.5 ppm, R^2^ = 0.998) barely outperforms Equation (16) (RMSE = 5.09 ppm, R^2^ = 0.997). As shown by Equation (22), a model of this complexity is also more vulnerable to overfitting (Figure A5).

The methane decay experiments highlight the importance of verifying machine-learning models beyond simply assessing the nominal model performance. It would not be possible to identify the overfitting of Equation (22) using the training results alone, and applying this model to field data would severely misrepresent the true methane concentration.

Of the tested model equations, Equation (16) (RMSE = 5.09 ppm, R^2^ = 0.997, complexity = 12) offered the best compromise between performance and complexity.

### 4.1. Comparison to Related Studies

Compared with a similar study using the Figaro NGM2611-E13 by Bastviken et al. [16], the study presented here covered lower temperatures (5–30 °C vs. 10–42 °C) and higher relative humidities (40–85% vs. 18–70%). This study trained models on over 50,000 data points, whereas Bastviken et al. used an average of 619–930 data points per sensor. The models tested by Bastviken et al. included temperature and humidity compensation but did not test interactions between predictor variables. In this study, several models which included interaction terms outperformed those which did not, highlighting the importance of considering them in future models.

Bastviken et al. used two approaches to determine the methane concentration: direct measurement using a reference sensor (a Los Gatos Research ultraportable greenhouse gas analyzer), or estimating the background methane concentration. If validated and performed with care, background methane concentration estimations may simplify sensor calibration, but they have the potential to introduce systematic errors which may be of a similar order of magnitude to the methane concentrations that the calibrated sensors are intended to measure. This issue is more easily circumvented by supplying sensors with a known concentration of methane during calibration, as was achieved with the use of a reference gas in this study.

Collier-Oxandale et al. [15] employed co-location of the methane sensors with reference instruments during field deployment. As stated by Collier-Oxandale et al., this approach exposes sensors to representative field conditions, rather than constraining them to more conventional laboratory settings, which typically control environmental conditions more strictly than real-world settings. However, much longer co-deployments may be required to obtain a broad range of conditions. If no high-emission or extreme events occur during the co-deployment phase, sensor models calibrated in this way may be poorly calibrated for these scenarios, being weighted towards typical field concentrations. As previously mentioned, this study also used time-based predictor variables in most of the presented calibration models. As such, calibrations based on these models would not be transferable to sensors deployed in a different location. Laboratory-based calibration with a methane concentration range which extends beyond the expected range of field values can be more readily generalised and is less biased towards “normal” diurnal cycles or environmental conditions.

Van den Bossche et al. [14] calibrated a TGS2611-E00 methane sensor across 15–30 °C, 40–80% relative humidity and 2–9 ppm methane. Methane concentration was recorded using a Picarro G2301 Cavity Ringdown Gas Analyzer. The linear fit used for the sensor calibration is a reasonable simplification for narrow ranges of methane concentrations but would underestimate higher methane concentrations. As in the study by Bastviken et al., van den Bossche et al. applied temperature and humidity compensation but neglected interactions between environmental conditions and methane concentration. Arguably, this is less crucial at low concentrations but should be investigated as a potential method for further improving the performance of low-concentration calibrations.

Due to the different concentration ranges used, it is difficult to make direct comparisons between the accuracy of different calibration approaches across these studies. However, a strong correlation between sensor response and true methane concentration below 300 ppm is consistently achieved, and the need to apply both temperature and humidity compensation is identified by all authors.

In the context of commodifying methane sensors for IoT applications, a shared limitation across all of these studies is the use of expensive reference sensors in the calibration approach. Such instruments often cost tens of thousands of pounds, placing them beyond the reach of many citizen scientists or smaller research groups. The calibration air-based method presented in this study offers an alternative low-cost approach; the estimated total cost of the calibration setup (vacuum pump, chamber, Arduino datalogger and calibration gases) is under GBP 500.

### 4.2. Limitations and Future Work

The TGS2611-E00 sensor incorporates a charcoal filter which improves the sensor selectivity by reducing the influence of other gases, such as ethanol and iso-butane. However, the sensor is also sensitive to hydrogen, making it less appropriate for detecting low methane levels in environments where hydrogen may also be present. This is less likely to be an issue in outdoor settings where hydrogen levels are typically much lower than methane levels, but the incorporation of additional sensors to measure the concentration of interference gases should be considered in relevant applications.

The variable warm-up period for the NGM2611-E13 sensors may pose a barrier to their usage in low-power applications; future work should investigate the effect of this warm-up period on intermittently powered NGM2611-E13 sensors.

## 5. Conclusions

Overall, the experiments presented show that the NGM2611-E13 methane sensors show a similar relationship between sensor resistance and methane concentration at methane concentrations below the response documented by the manufacturer for methane concentrations in the 300–10,000 ppm range. The presented trained models show promise for calibrating the NGM2611-E13 methane sensors at low methane concentrations across a range of temperature and humidity conditions. The relative performance of different model equations highlights the importance of considering the interaction between predictor variables. For example, the inclusion of a temperature and sensor voltage interaction term was shown to reduce model error and improve the correlation between the model prediction and the true methane concentration.

The presented calibration approach offers an efficient method for calibrating NGM2611-E13 methane sensors using only two pre-balanced calibration gas mixtures and without depending on a more expensive state-of-the-art reference sensor to measure the methane concentration. Data collected at additional intermediate methane concentration levels could be used to further refine these models. Likewise, calibrating the sensors over an even broader range of temperature and humidity conditions may be valuable for environmental monitoring settings.

The methane decay validation experiment presents an intuitive method for identifying inadequacies in calibration models that may not be obvious from the performance of models on training data. For example, spikes in predicted methane concentration that coincide with spikes in temperature clearly indicate insufficient temperature compensation.

This approach to calibrating gas sensors below their intended application concentration ranges may be extended to other low-cost sensors in the future, with the potential to broaden the range of pollutants that can be monitored by wireless sensor networks.

## Figures and Tables

**Table 1:** The model equations tested, where T is temperature (°C), H is relative humidity (%), $V_{out}$ is the sensor output voltage (V) and $C_{1}$–$C_{8}$ are constants to be optimised by the fitting algorithm.

No.	Predicted CH_4_ Equation
1	$C_{2}\cdot \exp\left(C_{3}\cdot V_{out}\right)$
2	$C_{1}+C_{2}\cdot \exp\left(C_{3}\cdot V_{out}\right)$
3	$C_{2}\cdot \exp\left(C_{3}\cdot V_{out}-C_{4}\cdot \ln\left(T+273.15\right)\right)$
4	$C_{1}+C_{2}\cdot \exp\left(C_{3}\cdot V_{out}-C_{4}\cdot \ln\left(T+273.15\right)\right)$
5	$C_{2}\cdot \exp\left(C_{3}\cdot V_{out}-C_{4}\cdot \ln\left(T+65\right)\right)$
6	$C_{1}+C_{2}\cdot \exp\left(C_{3}\cdot V_{out}-C_{4}\cdot \ln\left(T+65\right)\right)$
7	$C_{2}\cdot \exp\left(C_{3}\cdot V_{out}-C_{5}\cdot \ln\left(H\right)\right)$
8	$C_{1}+C_{2}\cdot \exp\left(C_{3}\cdot V_{out}-C_{5}\cdot \ln\left(H\right)\right)$
9	$C_{2}\cdot \exp\left(C_{3}\cdot V_{out}-C_{4}\cdot \ln\left(T+273.15\right)-C_{5}\cdot \ln\left(H\right)\right)$
10	$C_{1}+C_{2}\cdot \exp\left(C_{3}\cdot V_{out}-C_{4}\cdot \ln\left(T+273.15\right)-C_{5}\cdot \ln\left(H\right)\right)$
11	$C_{2}\cdot \exp\left(C_{3}\cdot V_{out}-C_{4}\cdot \ln\left(T+65\right)-C_{5}\cdot \ln\left(H\right)\right)$
12	$C_{1}+C_{2}\cdot \exp\left(C_{3}\cdot V_{out}-C_{4}\cdot \ln\left(T+65\right)-C_{5}\cdot \ln\left(H\right)\right)$
13	$C_{2}\cdot \exp\left(C_{3}\cdot V_{out}-C_{4}\cdot \ln\left(T+65\right)-C_{5}\cdot \ln\left(H\right)\right)-C_{6}\cdot \ln\left(\left(T+65\right)\cdot H\right)$
14	$C_{1}+C_{2}\cdot \exp\left(C_{3}\cdot V_{out}-C_{4}\cdot \ln\left(T+65\right)-C_{5}\cdot \ln\left(H\right)\right)-C_{6}\cdot \ln\left(\left(T+65\right)\cdot H\right)$
15	$C_{2}\cdot \exp\left(C_{3}\cdot V_{out}-C_{4}\cdot \ln\left(T+65\right)-C_{5}\cdot \ln\left(H\right)\right)-C_{7}\cdot \ln\left(\left(T+65\right)\cdot V_{out}\right)$
16	$C_{1}+C_{2}\cdot \exp\left(C_{3}\cdot V_{out}-C_{4}\cdot \ln\left(T+65\right)-C_{5}\cdot \ln\left(H\right)\right)-C_{7}\cdot \ln\left(\left(T+65\right)\cdot V_{out}\right)$
17	$C_{2}\cdot \exp\left(C_{3}\cdot V_{out}-C_{4}\cdot \ln\left(T+65\right)-C_{5}\cdot \ln\left(H\right)\right)-C_{8}\cdot \ln\left(\left(H\cdot V_{out}\right)\right)$
18	$C_{1}+C_{2}\cdot \exp\left(C_{3}\cdot V_{out}-C_{4}\cdot \ln\left(T+65\right)-C_{5}\cdot \ln\left(H\right)\right)-C_{8}\cdot \ln\left(\left(H\cdot V_{out}\right)\right)$
19	$C_{2}\cdot \exp\left(C_{3}\cdot V_{out}-C_{4}\cdot \ln\left(T+65\right)-C_{5}\cdot \ln\left(H\right)\right)-C_{7}\cdot \ln\left(\left(T+65\right)\cdot V_{out}\right)-C_{8}\cdot \ln\left(\left(H\cdot V_{out}\right)\right)$
20	$C_{1}+C_{2}\cdot \exp\left(C_{3}\cdot V_{out}-C_{4}\cdot \ln\left(T+65\right)-C_{5}\cdot \ln\left(H\right)\right)-C_{7}\cdot \ln\left(\left(T+65\right)\cdot V_{out}\right)-C_{8}\cdot \ln\left(\left(H\cdot V_{out}\right)\right)$

**Table 2:** Additional manually adjusted model equations.

No.	Predicted CH_4_ Equation
21	$C_{1}+C_{2}\cdot \exp\left(C_{3}\cdot V_{out}-C_{4}\cdot \ln\left(T+273.15\right)-C_{5}\cdot \ln\left(H+50\right)\right)-C_{6}\cdot \ln\left(H+50\right))\cdot \ln\left(V_{out}\right)$ $-C_{7}\cdot \ln\left(\left(T+273.15\right)\cdot V_{out}\right)-C_{8}\cdot \ln\left(T+273.15\right)\cdot \ln\left(H+50\right))$
22	$C_{1}+C_{2}\cdot \exp\left(C_{3}\cdot V_{out}-C_{4}\cdot \ln\left(T+273.15\right)-C_{5}\cdot \ln\left(H+0\right)\right)-C_{6}\cdot \ln\left(H+0\right))\cdot \ln\left(V_{out}\right)$ $-C_{7}\cdot \ln\left(\left(T+273.15\right)\cdot V_{out}\right)-C_{8}\cdot \ln\left(T+273.15\right)\cdot \ln\left(H+0\right))$

**Table 3:** A summary of the performance of the optimised model equations listed in Table 1, tested using the training data extracted from the Figaro datasheets [26].

Model No.	RMSE (PPM)	R^2^	Complexity
1	1.35 × 10^3^	0.724	3
2	1.27 × 10^3^	0.758	4
3	1.08 × 10^3^	0.823	6
4	912	0.876	7
5	735	0.918	6
6	725	0.922	7
7	1.21 × 10^3^	0.783	5
8	1.16 × 10^3^	0.801	6
9	935	0.870	8
10	849	0.894	9
11	238	0.992	8
12	231	0.992	9
13	238	0.992	12
14	263	0.990	13
15	196	0.994	12
16	224	0.993	13
17	196	0.994	11
18	197	0.994	12
19	198	0.994	15
20	224	0.993	16

**Table 4:** A summary of the performance of the optimised model equations tested using experimental data from 0–200 ppm methane concentrations.

Eq.	Predicted CH_4_ Equation	RMSE (ppm)	R^2^	Complexity
5	$C_{2}\cdot \exp\left(C_{3}\cdot V_{out}-C_{4}\cdot \ln\left(T+65\right)\right)$	55	0.684	6
6	$C_{1}+C_{2}\cdot \exp\left(C_{3}\cdot V_{out}-C_{4}\cdot \ln\left(T+65\right)\right)$	35.6	0.868	7
11	$C_{2}\cdot \exp\left(C_{3}\cdot V_{out}-C_{4}\cdot \ln\left(T+65\right)-C_{5}\cdot \ln\left(H\right)\right)$	19.2	0.962	8
12	$C_{1}+C_{2}\cdot \exp\left(C_{3}\cdot V_{out}-C_{4}\cdot \ln\left(T+65\right)-C_{5}\cdot \ln\left(H\right)\right)$	14.2	0.979	9
15	$C_{2}\cdot \exp\left(C_{3}\cdot V_{out}-C_{4}\cdot \ln\left(T+65\right)-C_{5}\cdot \ln\left(H\right)\right)-C_{7}\cdot \ln\left(\left(T+65\right)\cdot V_{out}\right)$	16	0.973	12
16	$C_{1}+C_{2}\cdot \exp\left(C_{3}\cdot V_{out}-C_{4}\cdot \ln\left(T+65\right)-C_{5}\cdot \ln\left(H\right)\right)-C_{7}\cdot \ln\left(\left(T+65\right)\cdot V_{out}\right)$	5.09	0.997	13
17	$C_{2}\cdot \exp\left(C_{3}\cdot V_{out}-C_{4}\cdot \ln\left(T+65\right)-C_{5}\cdot \ln\left(H\right)\right)-C_{8}\cdot \ln\left(\left(H\cdot V_{out}\right)\right)$	14.7	0.977	11
18	$C_{1}+C_{2}\cdot \exp\left(C_{3}\cdot V_{out}-C_{4}\cdot \ln\left(T+65\right)-C_{5}\cdot \ln\left(H\right)\right)-C_{8}\cdot \ln\left(\left(H\cdot V_{out}\right)\right)$	5.1	0.997	12
19	$C_{2}\cdot \exp\left(C_{3}\cdot V_{out}-C_{4}\cdot \ln\left(T+65\right)-C_{5}\cdot \ln\left(H\right)\right)-C_{7}\cdot \ln\left(\left(T+65\right)\cdot V_{out}\right)$ $-C_{8}\cdot \ln\left(\left(H\cdot V_{out}\right)\right)$	14.8	0.977	15
20	$C_{1}+C_{2}\cdot \exp\left(C_{3}\cdot V_{out}-C_{4}\cdot \ln\left(T+65\right)-C_{5}\cdot \ln\left(H\right)\right)-C_{7}\cdot \ln\left(\left(T+65\right)\cdot V_{out}\right)$ $-C_{8}\cdot \ln\left(\left(H\cdot V_{out}\right)\right)$	5.09	0.997	16
21	$C_{1}+C_{2}\cdot \exp\left(C_{3}\cdot V_{out}-C_{4}\cdot \ln\left(T+273.15\right)-C_{5}\cdot \ln\left(H+50\right)\right)-C_{6}\cdot \ln\left(H+50\right))\cdot \ln\left(V_{out}\right)$ $-C_{7}\cdot \ln\left(\left(T+273.15\right)\cdot V_{out}\right)-C_{8}\cdot \ln\left(T+273.15\right)\cdot \ln\left(H+50\right))$	4.5	0.998	23
22	$C_{1}+C_{2}\cdot \exp\left(C_{3}\cdot V_{out}-C_{4}\cdot \ln\left(T+273.15\right)-C_{5}\cdot \ln\left(H+0\right)\right)-C_{6}\cdot \ln\left(H+0\right))\cdot \ln\left(V_{out}\right)$ $-C_{7}\cdot \ln\left(\left(T+273.15\right)\cdot V_{out}\right)-C_{8}\cdot \ln\left(T+273.15\right)\cdot \ln\left(H+0\right))$	4.79	0.998	20

**Table 5:** Coefficients and related statistical measures for model Equation (16) following calibration with experimental data.

	Estimate	Standard Error	*t*-Statistic	*p*-Value
$C_{1}$	−1370	116.08	−11.80	4.2 × 10^−32^
$C_{2}$	4081.4	7.335	556.43	0
$C_{3}$	0.1109	0.0088	12.59	2.8 × 10^−36^
$C_{4}$	0.2152	0.0170	12.64	1.4 × 10^−36^
$C_{5}$	0.0808	0.0065	12.50	8.3 × 10^−36^
$C_{6}$	−0.0587	0.0047	−12.55	4.6 × 10^−36^

**Figure A1:**
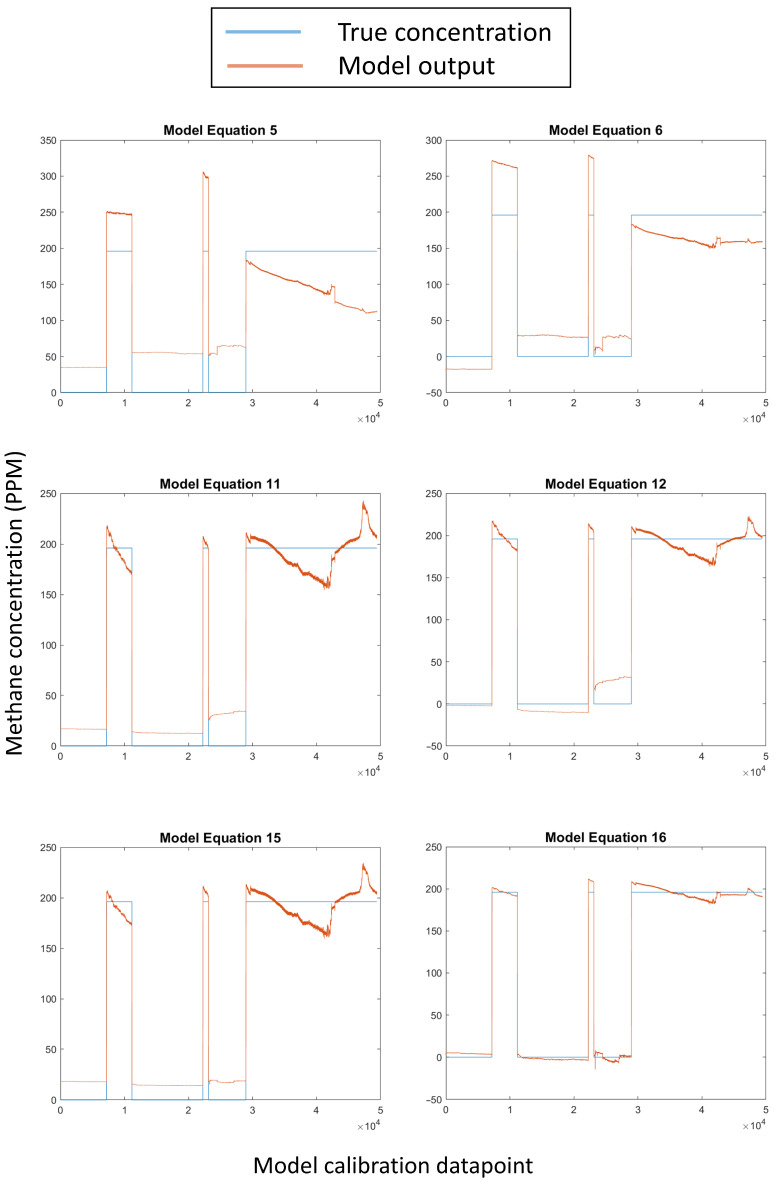
Trained models using Equations (5), (6), (11), (12), (15) and (16).

**Figure A2:**
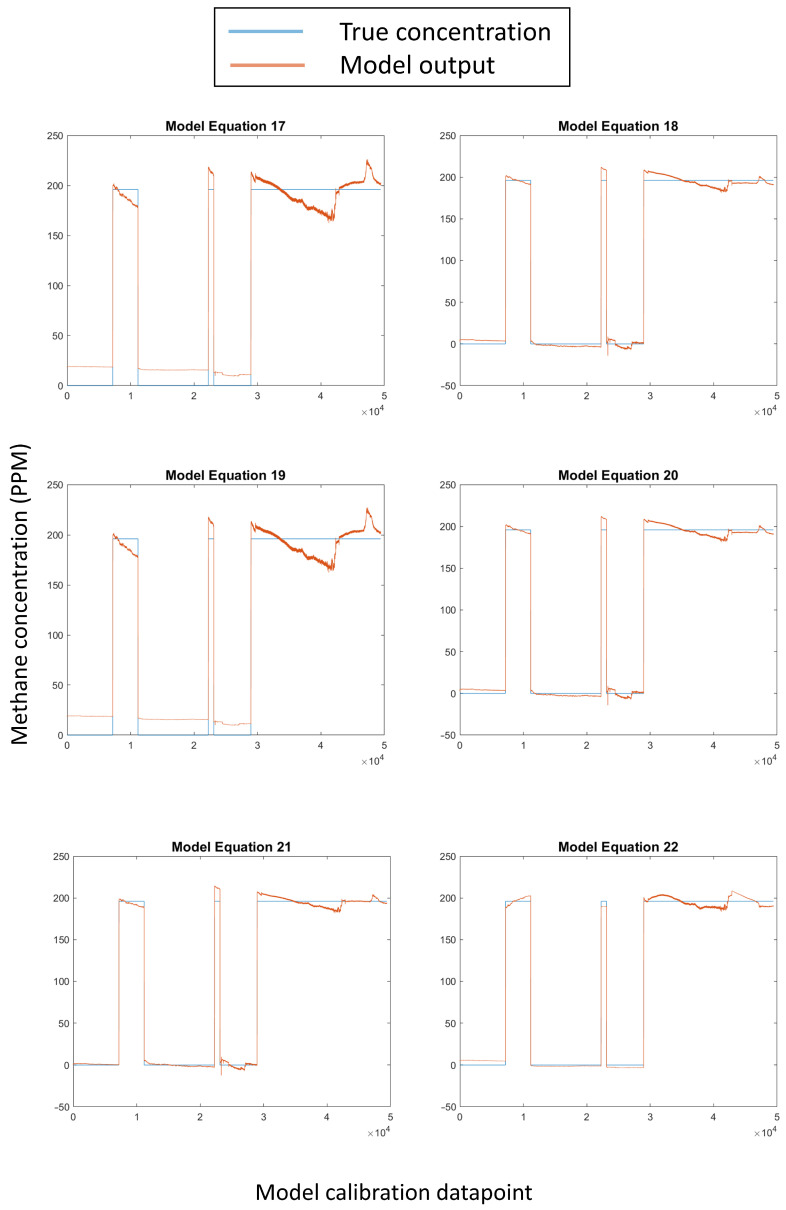
Trained models using Equations (17)–(22).

**Figure A3:**
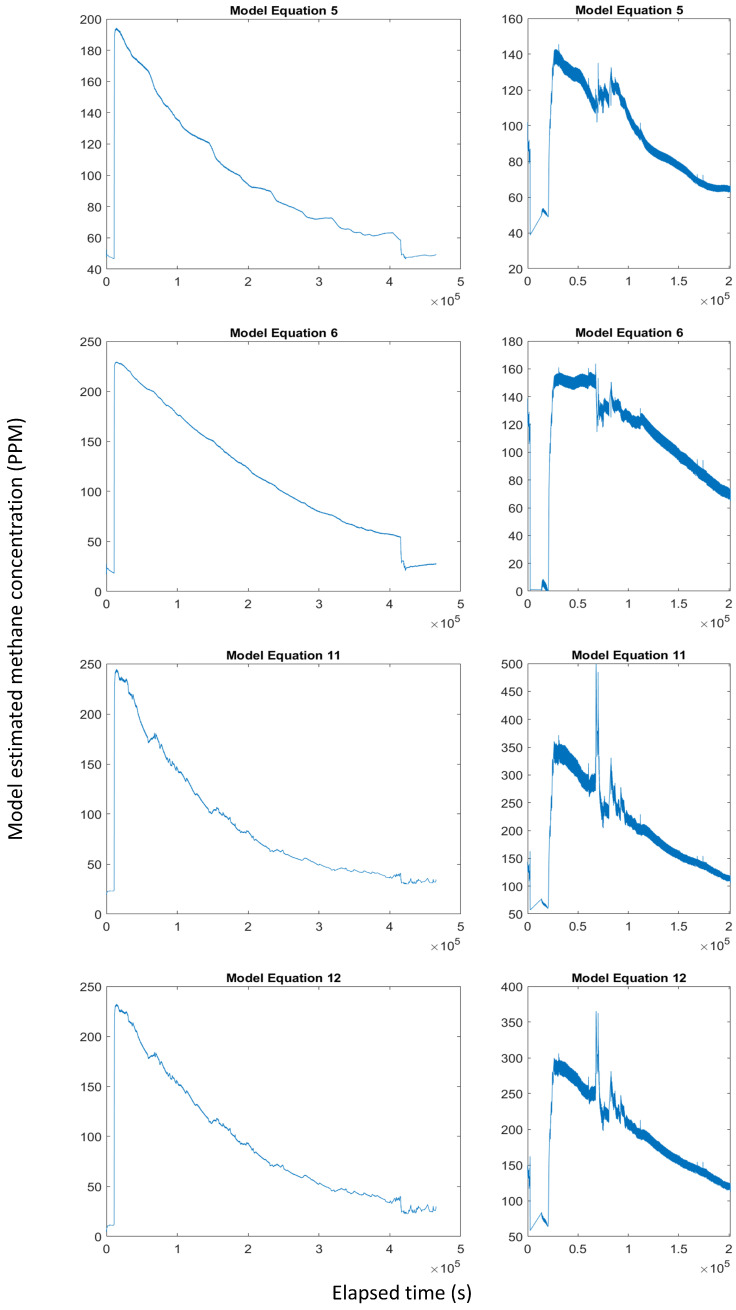
Estimated methane concentration from the decay experiment using model Equations (5), (6), (11) and (12).

**Figure A4:**
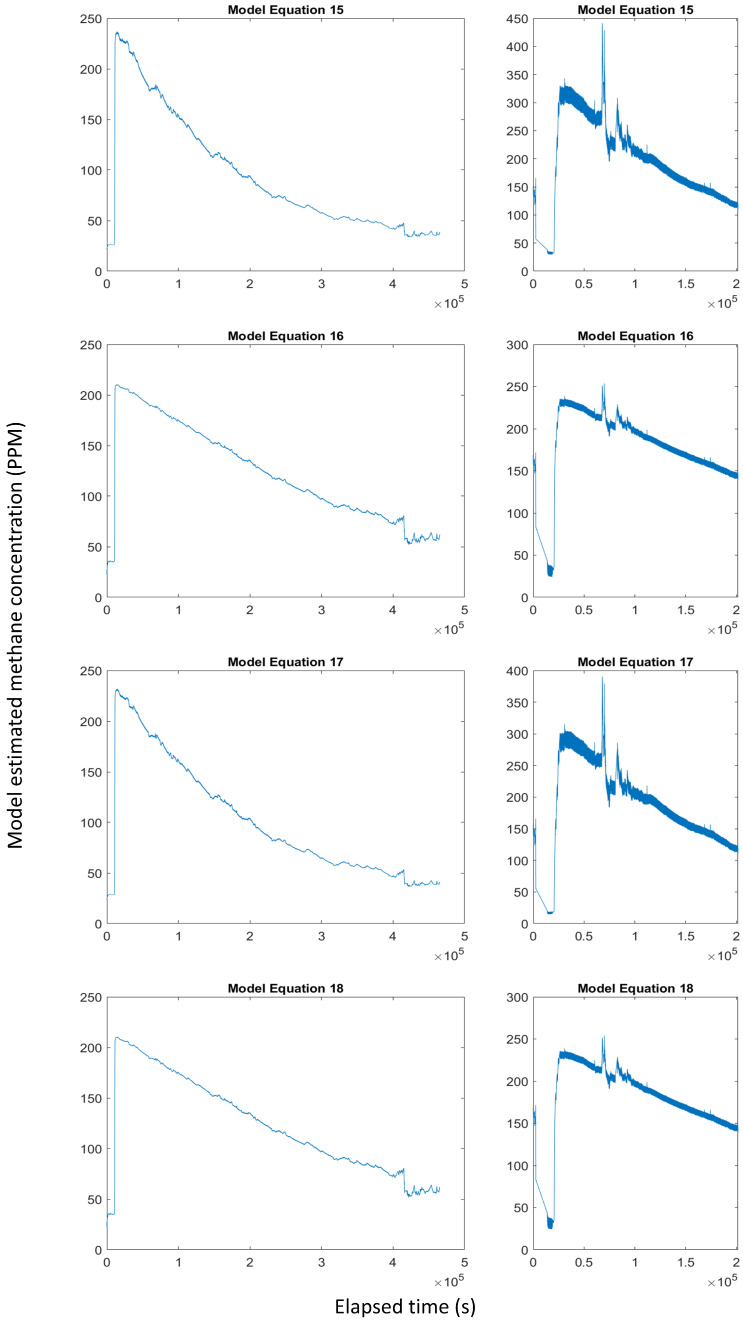
Estimated methane concentration from the decay experiment using model Equations (15)–(18).

## Data Availability

The calibration dataset can be found at: https://zenodo.org/doi/10.5281/zenodo.10405892 (accessed on 23 January 2024).

## Abbreviations

The following abbreviations are used in this manuscript:

CH_4_	Methane
PPM	Parts Per Million
RH	Relative Humidity
RMSE	Root Mean Squared Error
SD	Secure Digital
